# Research trends and hotspots of breast cancer management during the COVID-19 pandemic: A bibliometric analysis

**DOI:** 10.3389/fonc.2022.918349

**Published:** 2022-08-03

**Authors:** Peng-fei Lyu, Jing-tai Li, Tang Deng, Guang-Xun Lin, Ping-ming Fan, Xu-Chen Cao

**Affiliations:** ^1^ The First Department of Breast Cancer, National Clinical Research Center for Cancer, Key Laboratory of Cancer Prevention and Therapy, Key Laboratory of Breast Cancer Prevention and Therapy, Ministry of Education, Tianjin’s Clinical Research Center for Cancer, Tianjin Medical University Cancer Institute and Hospital, Tianjin Medical University, Tianjin, China; ^2^ Department of Breast Surgery, The First Affiliated Hospital of Hainan Medical University, Haikou, China; ^3^ Department of Interventional Radiology and Vascular Surgery, The First Affiliated Hospital of Hainan Medical University, Haikou, China; ^4^ Department of Orthopedics, The First Affiliated Hospital of Xiamen University, School of Medicine, Xiamen University, Xiamen, China; ^5^ The Third Clinical Medical College, Fujian Medical University, Fuzhou, China

**Keywords:** COVID-19, breast cancer, management, research hotspots, bibliometric analysis

## Abstract

**Background:**

The coronavirus disease 2019 (COVID-19) pandemic is disrupting routine medical care of cancer patients, including those who have cancer or are undergoing cancer screening. In this study, breast cancer management during the COVID-19 pandemic (BCMP) is reviewed, and the research trends of BCMP are evaluated by quantitative and qualitative evaluation.

**Methods:**

In this study, published studies relating to BCMP from 1 January 2020 to 1 April 2022 were searched from the Web of Science database (WoS). Bibliometric indicators consisted of publications, research hotspots, keywords, authors, journals, institutions, nations, and h-index.

**Results:**

A total of 182 articles investigating BCMP were searched. The United States of America and the University of Rome Tor Vergata were the nation and the institution with the most publications on BCMP. The first three periodicals with leading published BCMP studies were *Breast Cancer Research and Treatment, Breast*, and *In Vivo*. Buonomo OC was the most prolific author in this field, publishing nine articles (9/182, 4.94%). The co-keywords analysis of BCMP suggests that the top hotspots and trends in research are screening, surgery, rehabilitation, emotion, diagnosis, treatment, and vaccine management of breast cancer during the pandemic. The hotspot words were divided into six clusters, namely, screening for breast cancer patients in the pandemic, breast cancer surgery in the pandemic, recovery of breast cancer patients in the pandemic, motion effect of the outbreak on breast cancer patients, diagnosis and treatment of breast cancer patients in the pandemic, and vaccination management for breast cancer patients during a pandemic.

**Conclusion:**

BCMP has received attention from scholars in many nations over the last 3 years. This study revealed significant contributions to BCMP research by nations, institutions, scholars, and journals. The stratified clustering study provided the current status and future trends of BCMP to help physicians with the diagnosis and treatment of breast cancer through the pandemic, and provide a reference for in-depth clinical studies on BCMP.

## Introduction

Coronavirus disease 2019 (COVID-19), caused by severe acute respiratory syndrome coronavirus 2, has significantly affected >220 million individuals worldwide (1). The susceptibility to the adverse effects caused by COVID-19 has received huge amounts of global attention, due to the potentially increased vulnerability of COVID-19-induced mortality (2). During the pandemic, the management of cancer patients has changed significantly, which consists of delayed breast cancer screening, untimely treatment and follow-up, and breast cancer recovery after infection with COVID-19 (3–8). The true effect of the COVID-19 pandemic on patient outcomes remains unknown, though most of the changes were reasonable responses to the current healthcare emergency (3). Breast cancer is one of the most common malignancies in women worldwide, and breast cancer has surpassed lung cancer as the most commonly diagnosed cancer, with an estimated 2.3 million new cases within a year (9, 10). Accordingly, there is an urgent need to find research on breast cancer management in pandemics for breast cancer patients. Bibliometric is a method of quantitative analysis, which employs co-keyword and co-citation analysis of previous studies to facilitate the identification of popular themes and emerging trends in all study fields (11, 12). Thus, many scholars have performed bibliometric analysis on diseases (13–16), using CiteSpace, VOSviewer, and Bibliographic Items Co-occurrence Matrix Builder (BICOMB) for analysis and visualization (17–19). Nevertheless, there have been no bibliometric studies about BCMP throughout the COVID-19 pandemic. Thus, through our study, research hotspots and future directions in this field were highlighted, providing a reference for in-depth clinical practice related to BCMP.

## Methods

### Systematic search strategy

This study was not approved by an institutional committee since the relevant public data were retrospectively reviewed. Articles were searched from the Web of Science (WoS) database. The literature between 1 January 2020 and 30 April 2022 was reviewed. The time frame of the study was from the outbreak of the COVID-19 to the present. Search phrases included (TI=(“neoplasm of the breast” OR “breast neoplasm” OR “carcinoma breast” OR “carcinoma of the breast” OR “breast cancer” OR “cancer of the breast” OR “breast cancer”)) AND TI=(“SARS-COV2” OR “Severe Acute Respiratory Syndrome Coronavirus-2” OR “SARS coronavirus 2” OR “2019 novel coronavirus” OR 2019-nCoV OR SARS-CoV-2 OR “coronavirus disease 2019” OR “coronavirus 2019” OR “COVID 19”) AND PY= (2012–2021)) AND DT=(Article)). Original articles were only incorporated; letters, editorial material, and reviews were excluded. A total of 182 articles were correlated with our topics. Two researchers verified that these publications matched the themes of this study. Any differences of opinion were discussed until a consensus was reached.

### Data analysis

CiteSpace, R language, and VOSviewer were utilized for creating data tables and visual knowledge graphs. CiteSpace is largely based on co-citation analysis and pathfinder network scaling to investigate the articles on a particular subject, which allows users to find the vital development and knowledge turning point in the discipline history (20). VOSviewer is a tool to create maps based on network, bibliographic, or text data (21). BCMP was analyzed with the use of the R-based Biblioshiny app, thus creating a web interface for bibliometrics (https://bibliometrix.org/) (22). Results are based on the qualitative and quantitative investigation according to the numbers of publications, nations, h-index [a valid and reliable indicator for academic assessment (23)], keywords, hotpots, co-occurrence status, citations, authors, journals, and institutions. The degree of communication in this field was partly based on surveys of co-authors. The links between the visualization knowledge maps among nodes showed the cooperative ties. The size of the circle represents the amount of relevant domain volume.

Bibliographic Item Co-Occurrence Matrix Builder (BICOMB) is available freely online (23, 24). Next, with the use of the software “gCLUTO”, version 1.0 (Graphical CLUstering TOolkit, a graphical front-end in terms of the CLUTO data clustering library, proposed by Rasmussen, http://glaros.dtc.umn.edu/gkhome/cluto/gcluto/download), a binary matrix was built in accordance with BICOMB based on commonplace significant MeSH terms representing the rows and with source articles representing the columns in terms of further biclustering (24). Parameters of biclustering in gCLUTO were set according to those appropriate for biclustering analysis based on articles (25). Repeated bisection was selected for the clustering method, cosine for the similarity function, and *I* ^2^ for the clustering criterion function. To distinguish the optimal number of clusters, the biclustering with different cluster numbers was rerun (26). With the use of matrix visualization as well as mountain visualization, we presented the biclustering results achieved by the matrix of extensive major keywords-source articles (27). The basic framework of research hotspots of BCMP was generated and studied based on the semantic relationship among hotspot words and the content of the typical paper in the respective cluster.

## Results

### Current status

After screening, 182 articles on the topic of BCMP during the COVID-19 pandemic were acquired from the WOS database in less than 3 years, particularly in 2021 with 104 articles accounting for 57.1% of the total literature, thus significantly contributing to this study.

### Analysis of nations and institutions

**Figure 1:**
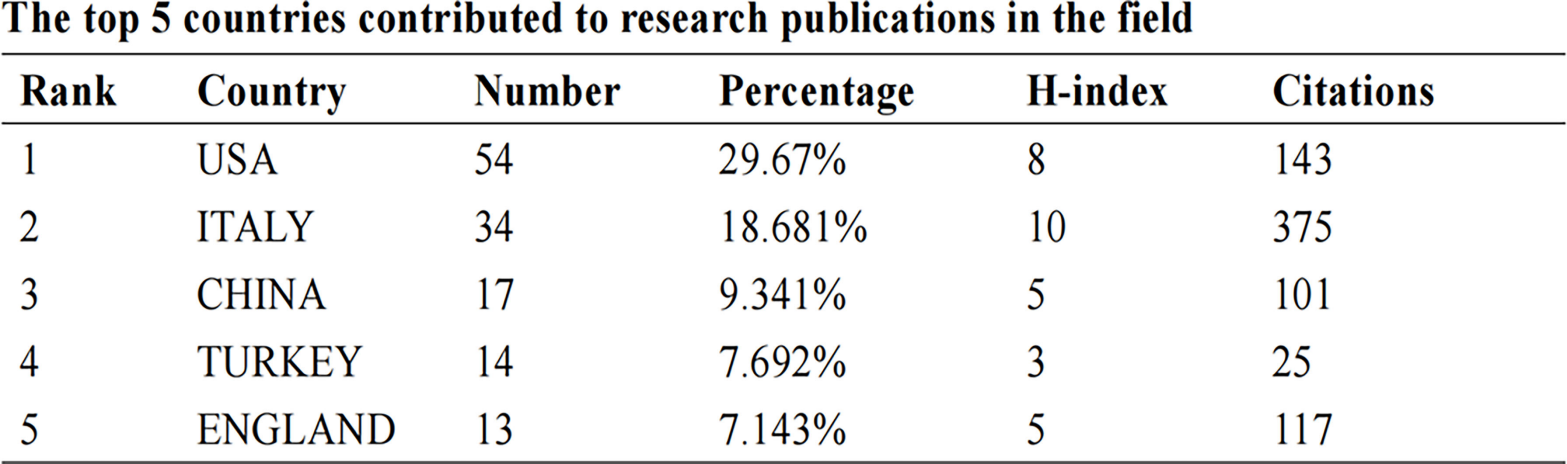
The top five countries that contributed to research publications in the field.

**Figure 2:**
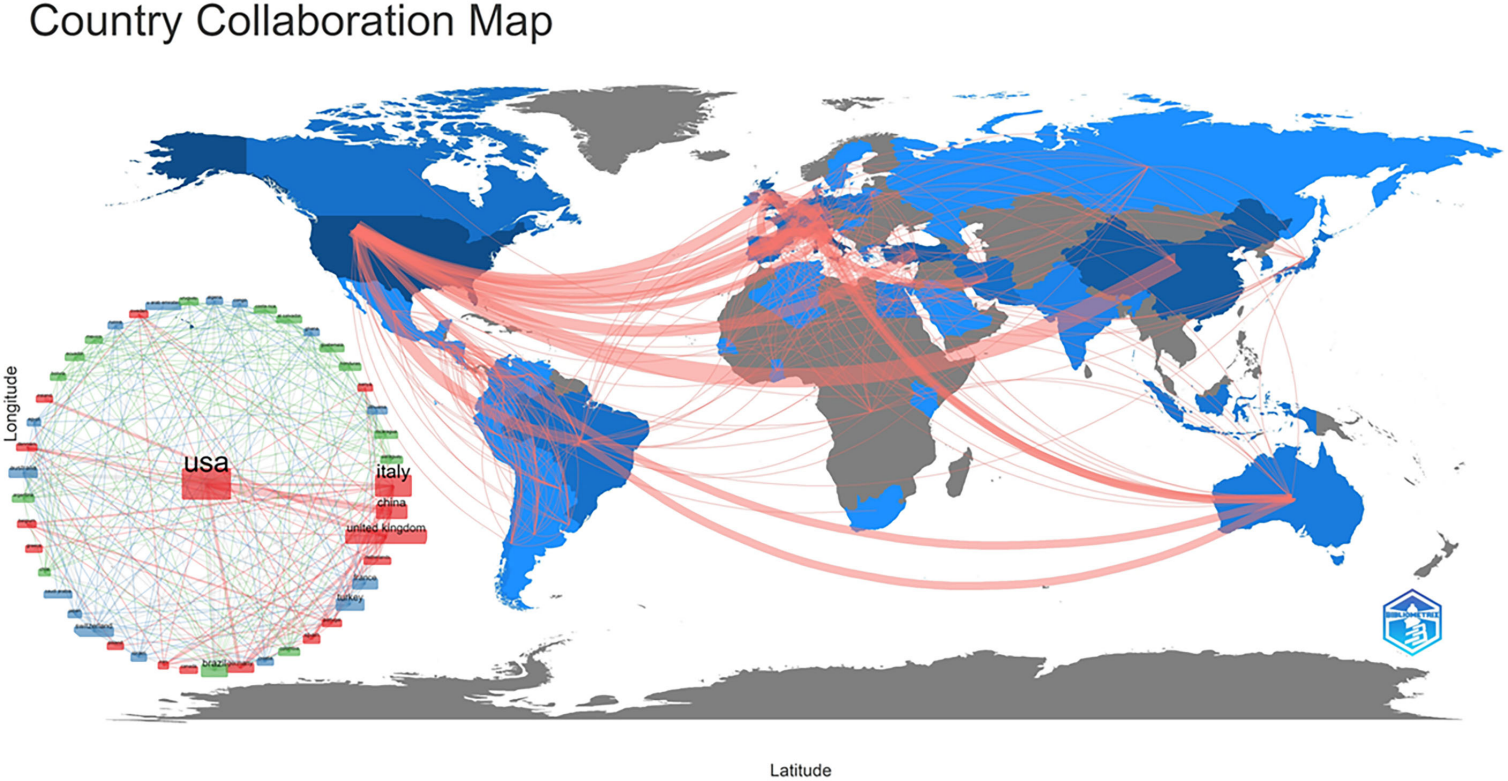
The country collaborative map in the field of BCMP.

**Figure 3:**
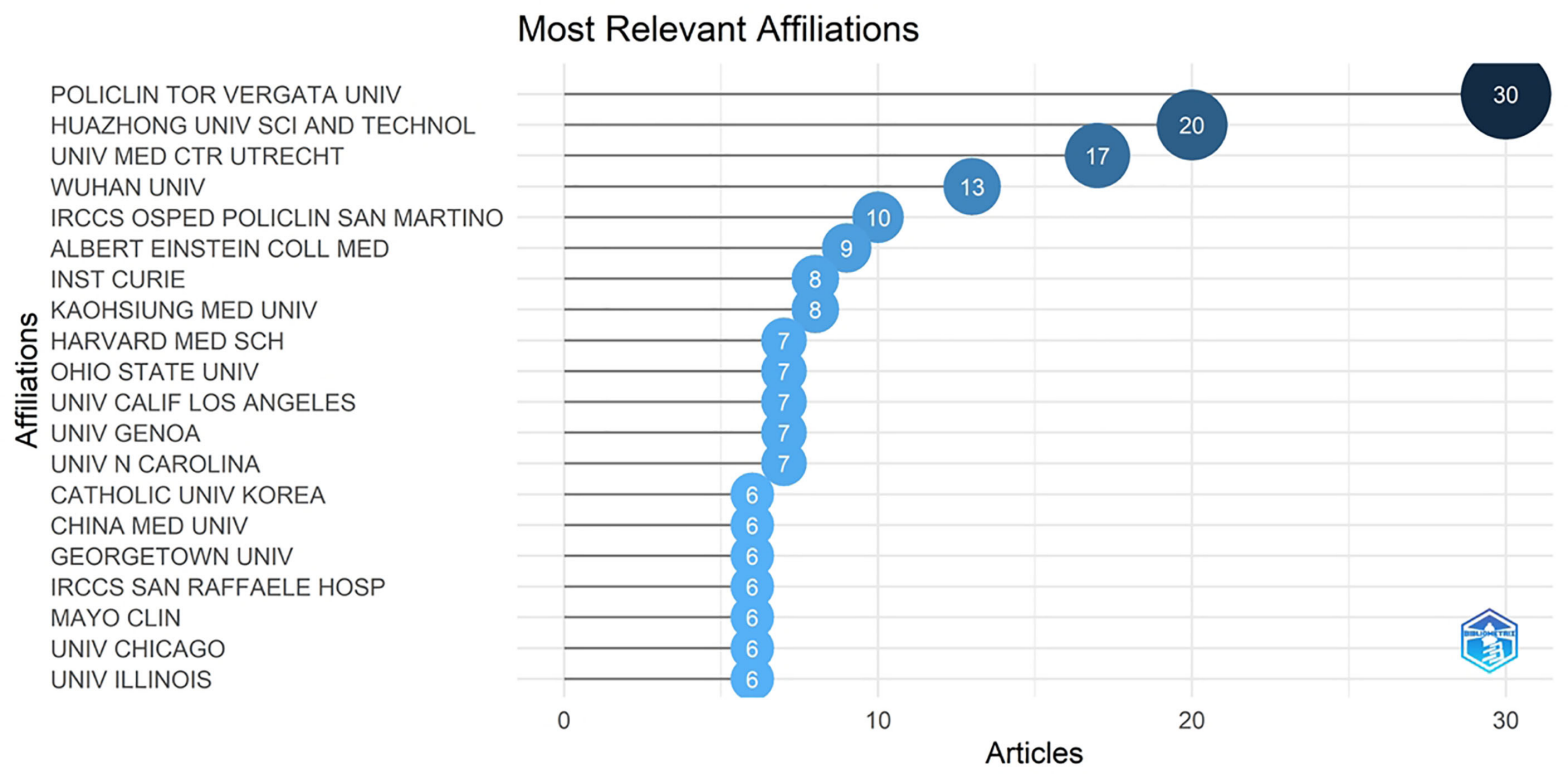
The most relevant affiliations linked to articles of BCMP (top 20).

**Figure 4:**
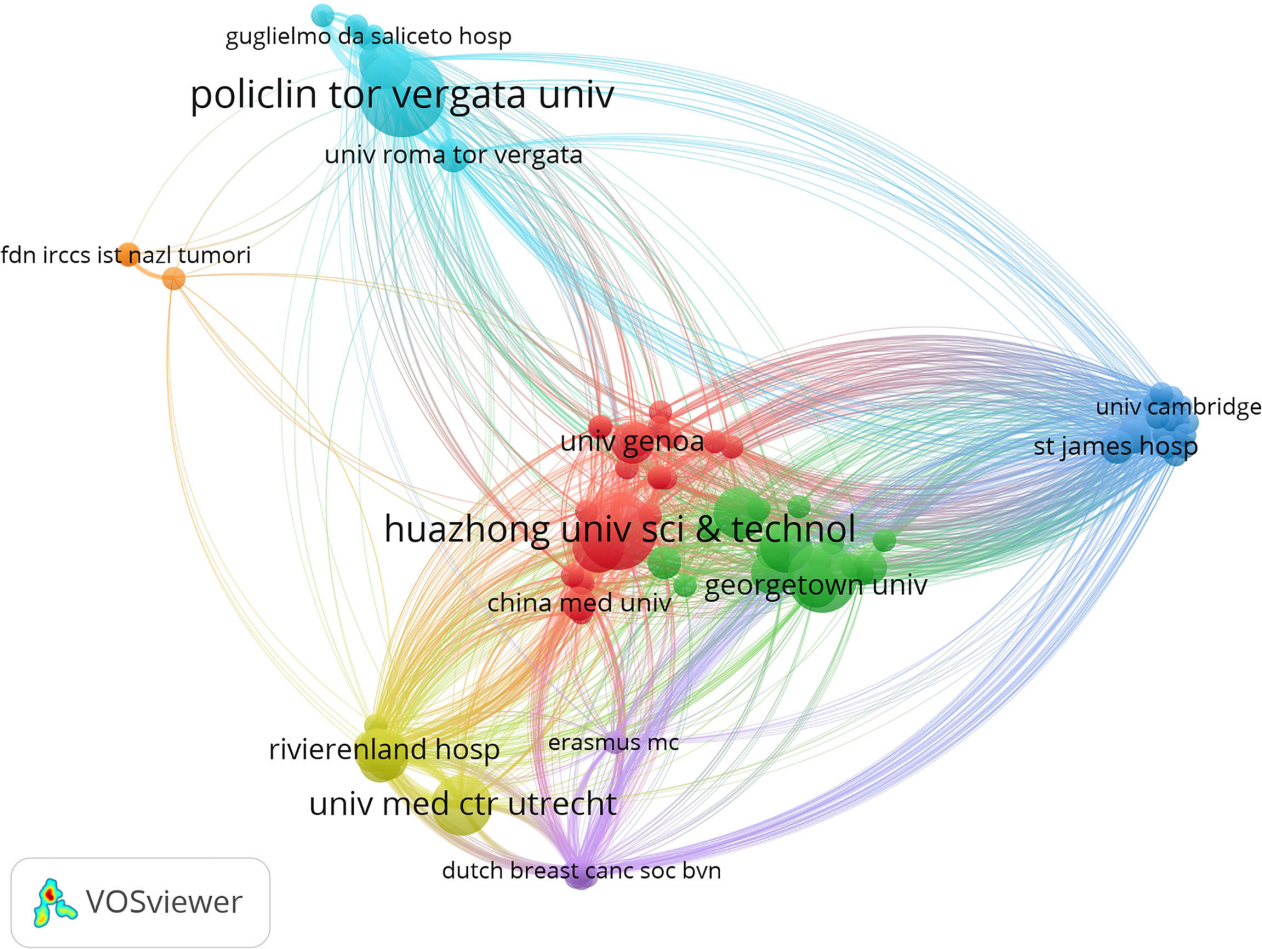
The map of the institution’s collaborative network.

A total of 60 nations contributed to breast cancer management during the COVID-19 pandemic in the study period. The United States of America had the largest number of articles (54 of 182 [29.67%]), followed by Italy (34 articles [18.68%]), P.R. China (17 articles [9.34%]), Turkey (14 articles [7.69%]), and England (13 articles [7.14%]) (**Figure 1**). Italy achieved the maximum h-indexes (10), followed by the United States of America, China, Turkey, and England (**Figure 1**). The collaboration world map shows the number of publications, with darker colors representing more papers; the number of connecting lines represents the amount of cooperation between nations (**Figure 2**). The United States of America, China, and the UK, with the United States of America at the core, are working and communicating tightly in terms of BCMP. **Figure 3** presents the prolific institutions in BMCP. Policlinico Tor Vergata University published 30 articles, Huazhong University of Science and Technology published 20 articles, and University Medical Center Utrecht published 17 articles. Policlinico Tor Vergata University is the most relevant institution linked to BCMP because it has the largest number of documents (**Figure 3**). **Figure 4** presents a map of the institution’s collaborative network related to BCMP. The same color means that the institution is from the same nation. Larger circles mean more articles were published. More connecting lines means more collaboration. The connecting line means cooperation, showing a centralized distribution and good collaboration among the above institutions. As depicted in **Figure 4**, Huazhong University of Science and Technology, University Medical Center Utrecht, and Policlinico Tor Vergata University cooperated closely in the field of BCMP.

### Analysis of journals

**Figure 5:**
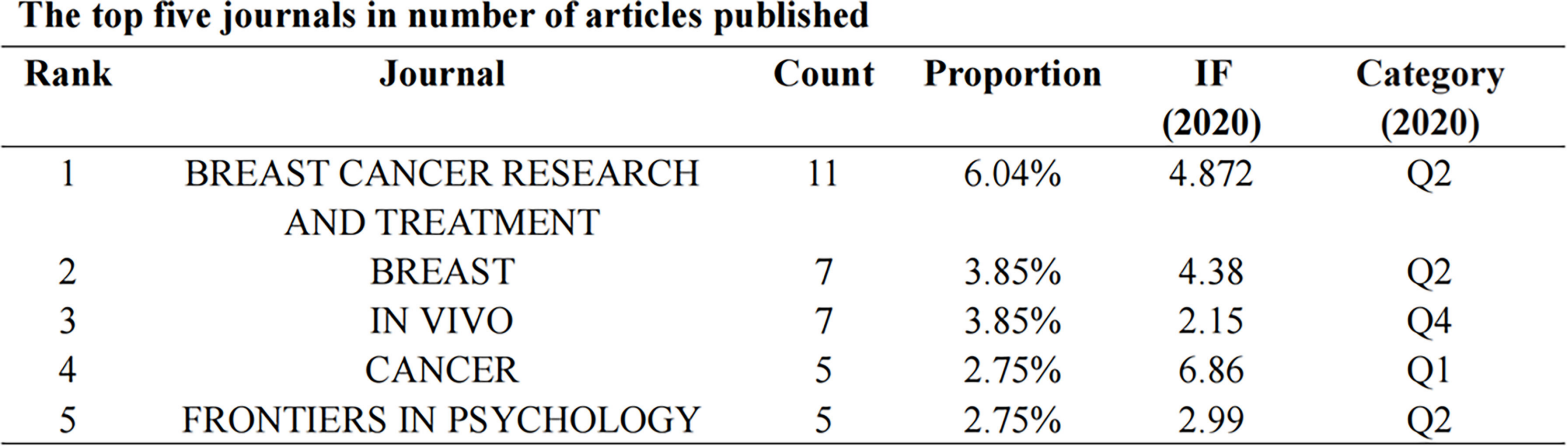
The top five journals in number of articles published.

Articles regarding BCMP were published in 101 science journals. The top five journals consisted of *Breast Cancer Research and Treatment*, with 11 papers (6.04%); *Breast*, with 7 (3.84%); the *In Vivo*, with 7 (3.84%); *Cancer*, with 5 (2.74%); and the *European Journal of Breast Health*, with 5 (2.74%) (3.75%; **Figure 5**). The average impact factor of the journals was about 4, with most belonging to Q2.

### Analysis of authors and references

**Figure 6:**
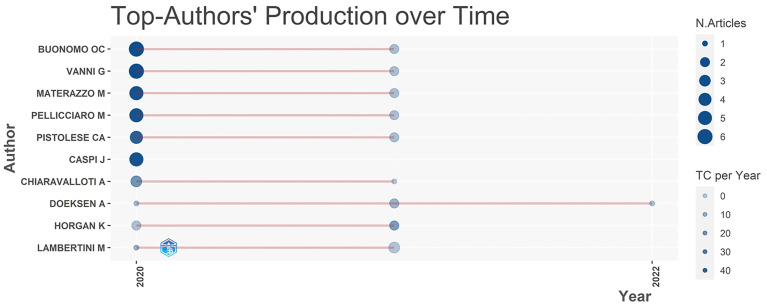
The top author’s production over time.

Buonomo OC was the most prolific author in this field, publishing nine articles (9/182, 4.94%), followed by Vanni G, Matarazzo M, and Pellicciaro M, who published eight articles (4.40%) and were cited over 30 times. Interestingly, all of them achieved the same h-index (h-index = 6) and came from the same institution (University of Rome Tor Vergata) and nation (Italy). The top 10 authors have a steady output and total citation in the 2 years. A list of the top authors’ productions over time is shown in **Figure 6**. The size of the dark blue circles represents the number of publications by the author; the size of the light blue circles represents how many times the article has been cited. Thus, **Figure 6** shows the evolution of publications and citations over time for highly productive authors. Authors who have published prolific articles are also highly cited authors.

The top 10 papers have been cited 465 times (**Supplementary Table S1**). The greatest and smallest number of citations for a particular article were 110 and 24. Four articles were from Italy, three from the UK, and one from China, France, and the United States of America.

### Analysis of keywords

**Figure 7:**
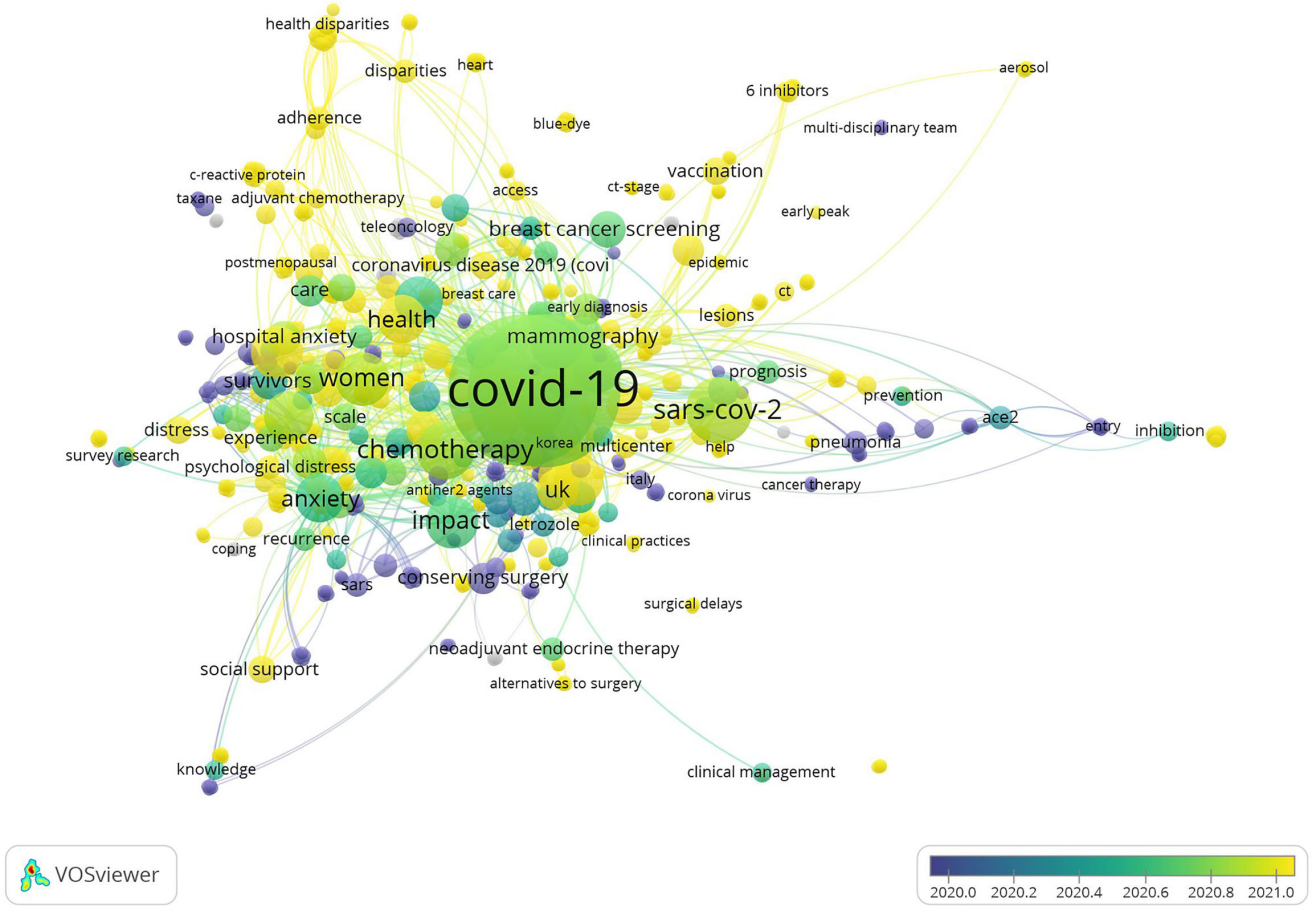
The co-occurrence map of keywords.

**Figure 8:**
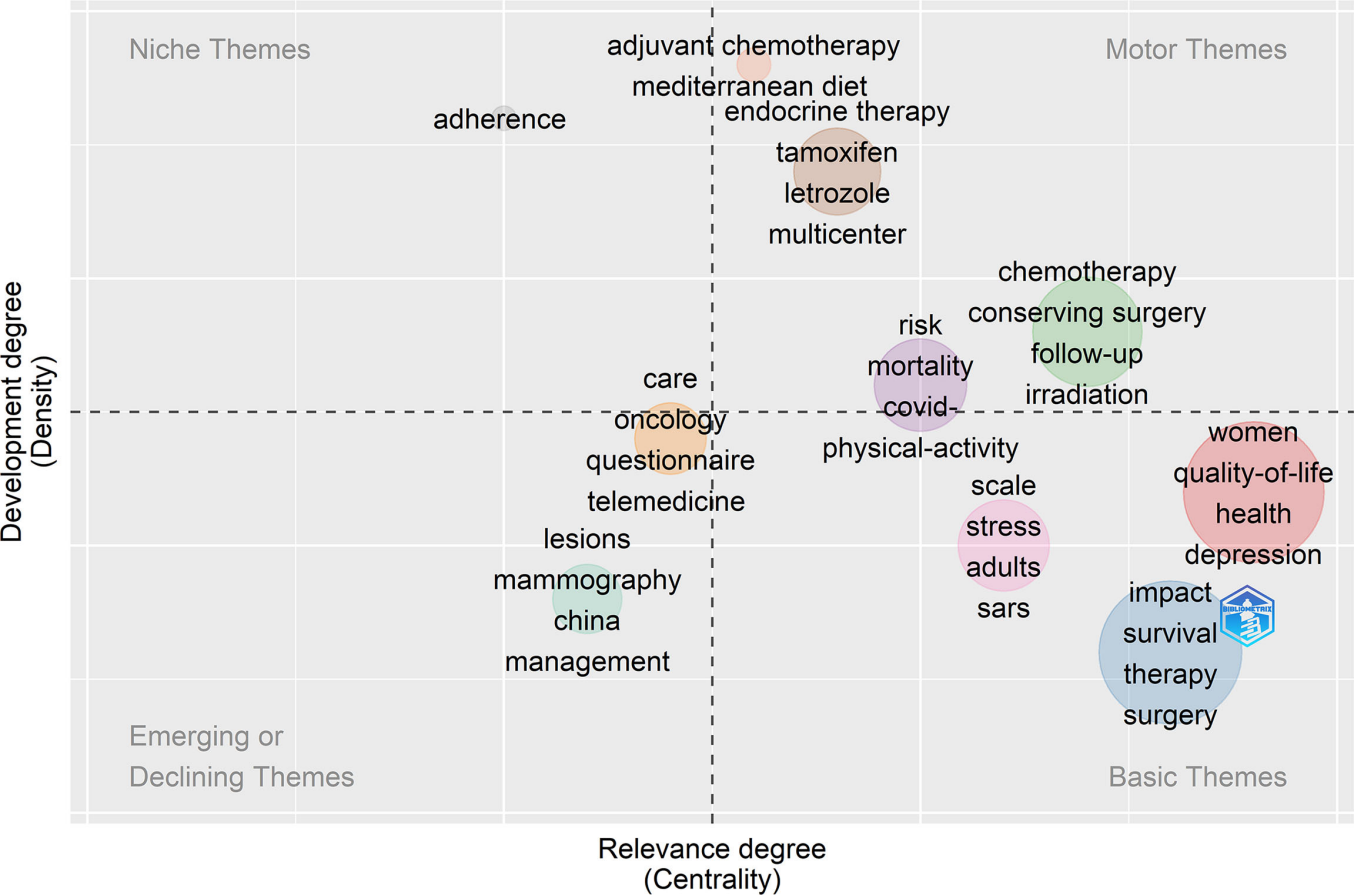
The thematic map of keywords plus.

With the use of high-frequency keywords to identify the hotspots of research, these and other vital issues can be effectively determined. A total of 380 keywords were extracted based on BICOMB from the 182 publications. The frequency of 2 was defined as high-frequency keywords, and 60 keyword matrixes were classified. The 10 most frequent keywords consisted of COVID-19, breast cancer, quality of life, women, impact, therapy, diagnosis, survival, health, and surgery. The keywords co-occurrence map shows that larger nodes had larger keyword weights, and the linkage of keywords represents simultaneous appearance in one document (**Figure 7**). The different colors in the co-occurrence chart can be observed, the dark color represents the keywords appearing earlier, and the light color represents the keywords appearing recently (**Figure 7**). The development direction of BCMP was analyzed according to the thematic map (**Figure 8**). The abscissa is the correlation degree of centrality, and the ordinate is the development degree of hotspot keywords. It is suggested that the basic themes are emotional influence, quality of life, and treatment. The mainstream themes are chemotherapy, neoadjuvant therapy, endocrine therapy, radiotherapy, breast-conserving surgery, and follow-up. The decline theme is questionnaire, telemedicine, and mammography.

### Cluster analysis of research hotspots

**Figure 9:**
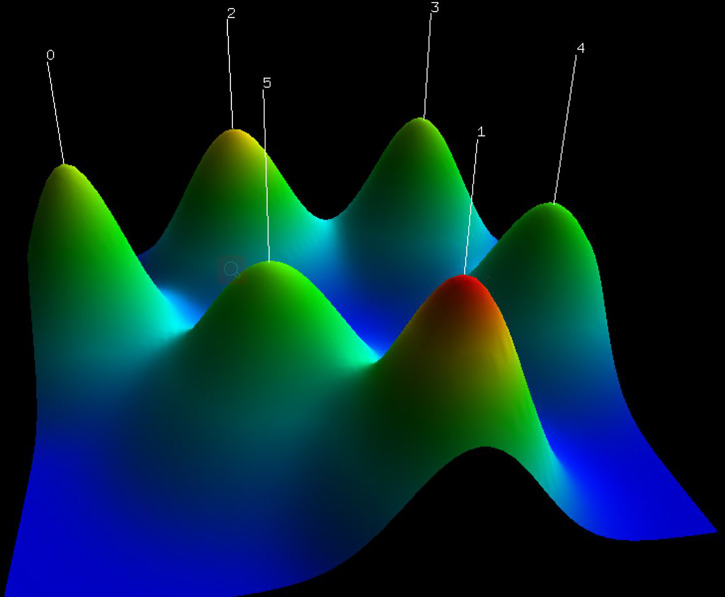
The visualized mountain map of the keywords: Cluster 0: Screening for breast cancer patients during the pandemic; Cluster 1: Breast cancer surgery in the pandemic; Cluster 2: Recovery of breast cancer patients during the pandemic; Cluster 3: Motion impact of the outbreak on breast cancer patients; Cluster 4: Diagnosis and treatment of breast cancer patients during the pandemic; Cluster 5: Clinical vaccine management of breast cancer patients during a pandemic.

**Figure 10:**
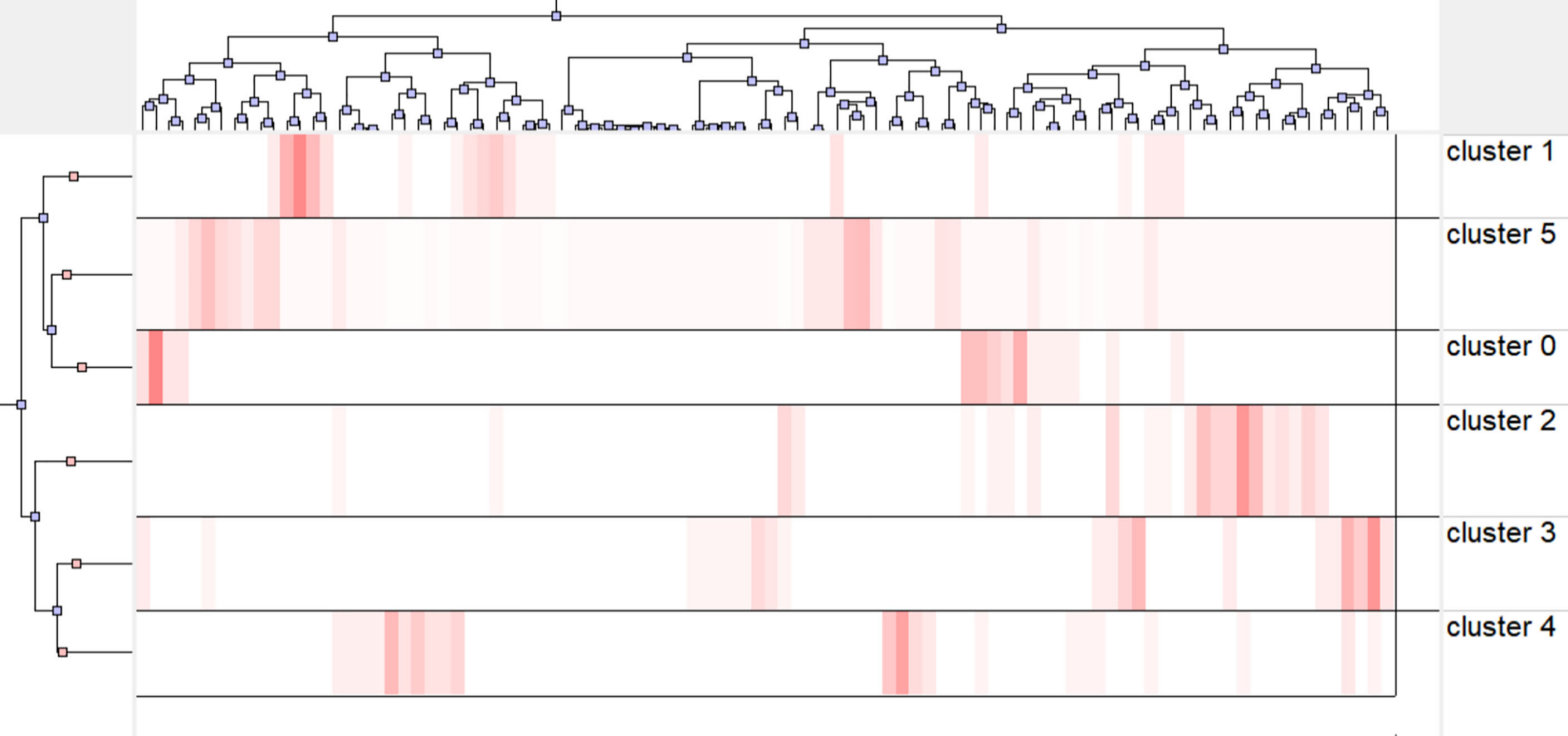
The visualized heat map linked to data matrix.

Subsequently, similar categories of keywords are assigned to the same cluster using gCLUTO for biclustering analysis. The above clusters revealed the vital research field and critical research content. The substantial number represents the cluster number within the visualized mountain map (**Figure 9**). The mountain volume was directly proportional to the keyword number in the cluster. In addition, a proportional relationship was found between height and within-group similarity. The red spikes represent good intraclass similarity, the yellow and green spikes represent average intraclass similarity, and the blue proxy variant shows low intraclass similarity. The distance between the peaks can be used to evaluate the similarity between clusters. The six small mountains were relatively independent and distributed, suggesting a significant clustering effect. **Figure 10** presents the visualized heat map linked to the keyword matrix. Rows represent published literature and columns represent cluster numbers. The colors stand for values within the initial data matrix. In general, color depth stands for the values in the initial data matrix. The white area stands for a value approaching zero. The deepened red area can represent a significant value. The major keywords for the six clusters are presented in **Supplementary Table S2**. The greater the internal similarity, the better the clustering; the smaller the external similarity, the better the clustering. Six research clusters in the field of BCMP were demonstrated by identifying the semantic connection in words with a high frequency and their source articles:

Cluster 0: Screening for breast cancer patients in the pandemic

Cluster 1: Breast cancer surgery in the pandemic

Cluster 2: Recovery of breast cancer patients in the pandemic

Cluster 3: Emotion effect of the outbreak on breast cancer patients

Cluster 4: Diagnosis and treatment of breast cancer patients in the pandemic

Cluster 5: Clinical vaccination management for breast cancer patients during the pandemic

## Discussion

The COVID-19 pandemic has caused major disruption to healthcare systems and professionals around the globe (28). Numerous experts provide recommendations to prepare for the effect of the COVID-19 pandemic on breast cancer patients and propose suggestions in terms of the method of triaging, prioritizing and organizing medical treatment, radiation, surgeries, and diagnoses (28–30).

The BCMP publications between 2020 and 2022 were investigated with the use of information visualization methods. A total of 421 BCMP-related articles were identified. Furthermore, 182 original articles were finally studied by de-duplicating verification, excluding reviews, conference articles, and letters.

The highest number of articles was from the United States of America. Although Italy has not published the most articles, it achieved the largest h-index. The reason may be that the pandemic situation in Italy was serious in the early stage of the pandemic, and more scholars have studied this field. University of Rome Tor Vergata published 30 papers, and Huazhong Universality of Science Technology published 20 articles. There is a certain amount of cooperation between the above nations or institutions. This analysis revealed that a considerable number of scholars and institutions have been concerned with BCMP over the past 3 years and explored corresponding solutions.

During the pandemic, doctors may be more concerned about breast cancer diagnosis, treatment, and quality of life. Since the pandemic can lead to quarantines and lockdowns, the above can cause delays in breast cancer-related diagnosis and treatment (31–33).

We analyzed the development direction of BCMP through the thematic map, which was derived from keywords plus, thus providing a more comprehensive view of trends in the field. The mainstream themes include chemotherapy, neoadjuvant therapy, endocrine therapy, radiotherapy, breast-conserving surgery, and follow-up, which are likely to be further developed in the future as it has a high level of development and relevance.

### Cluster 0: Screening for breast cancer patients in the pandemic

Pandemic-associated deficits in the number of breast examinations have been declining with time. The interrupted time series investigation demonstrated smaller frequencies of breast biopsy, diagnostic mammography, as well as screening mammography after the outbreak (34). The need for a modern, flexible national health system for making up for new challenges generated by further emerging pandemics has increased due to the COVID-19 pandemic (32, 35).

Furthermore, there may be a viable approach (36) that divides breast cancer screening into four types, namely, non-COVID-19 patients, confirmed COVID-19 in asymptomatic screening patients, suspected COVID-19 with symptomatic or confirmed breast cancer, and confirmed COVID-19 with symptomatic or confirmed breast cancer. Through the above approach, none of the medical staff or paramedics involved in the screening were infected (36).

### Cluster 1: Breast cancer surgery in the pandemic

Breast cancer surgery can be safely carried out and integrated with a stringent protocol for reducing COVID-19 exposure and transmission, despite the pressures associated with the COVID-19 pandemic (37). During the pandemic, scholars from Turkey considered administering neoadjuvant systemic therapy in patients with luminal A-like, HER2-positive, small-size triple-negative, and node-negative tumors until the conditions were improved by surgical treatment (38). A study from South Korea suggested that the prognosis of patients with delayed surgeries did not seem to change compared with patients who proceeded with their surgeries (39). Some doctors from China suggested that for early-stage breast cancer, especially stage I, surgical treatment should be performed within 30 days if conditions permit (40). To minimize the delay of treatment during the pandemic, Vanni et al. suggested that multi-disciplinary treatment (MDT) should triage patients and schedule surgical procedures to optimize the allocation of the limited resources to urgent cases (41). Nevertheless, some scholars suggested that complex reconstruction surgeries should be delayed in areas where the pandemic is not well controlled (28, 42, 43) due to the extended hospital stay for complex reconstructive surgery and the possible complications.

### Cluster 2: Recovery of breast cancer patients in the pandemic

A paper suggested that the treatment of ACEIs (angiotensin-converting enzyme ACE inhibitors) in Luminal A breast cancer might facilitate tumor progression (44). Jiang et al. suggested that breast tumor tissues can be further reduced at ACE2 expression level (angiotensin-converting enzyme 2) after SARS-CoV-2 infection, which further deteriorates immune infiltration and worsens the prognosis of luminal B breast cancer after SARS-CoV-2 infection (45).

Moreover, Mella-Abarca suggested that telerehabilitation may take on a great significance in people with breast cancer during the pandemic (46). It comprises a phone call, an individual video call using a mobile device (computer or smartphone), or a group video call, which is dependent on the convenience and availability of the individual’s devices (e.g., implementation of the model, prevention of lymphedema, lymphedema, and pre-surgical evaluation for breast cancer) (46).

During the COVID-19 pandemic, Okechukwu et al. suggested that cancer patients should exercise at home on a tele-supervised home-based exercise oncology platform tailored by a physician and certified clinical exercise physiologist based on their preferences, contraindications, exercise tolerance, current clinical status, medical history, and cardiorespiratory fitness/functional capacity, instead of exercising within an indoor public fitness facility or outdoor spaces to curb the risk of COVID-19 infection and cardiovascular events (47).

### Cluster 3: Emotion effect arising from the outbreak on breast cancer patients

During the pandemic, many breast cancer patients experienced many stressors related to more significant anxiety, depression, fear of cancer recurrence (FCR), and insomnia (48). Simultaneously, the quality of life of breast cancer patients was adversely affected (49). It is imperative to have conversations (phone or video) with breast cancer survivors about mental health and provide accessible services. Moreover, Papautsky et al. suggested that cancer patients should be trained with stress management strategies to acquire skills to manage their stress and prevent the adverse consequences of stress (50, 51).

### Cluster 4: Diagnosis and treatment of breast cancer patients in the pandemic

Some physicians suggested classifying people at risk of breast cancer and trying to diagnose them as early as possible, while those at low risk should be observed and followed up at home (52). The use of chemotherapeutic agents with low side effects is recommended for patients with postoperative adjuvant chemotherapy (53, 54).

Endocrine treatments [tamoxifen, aromatase inhibitors, and luteinizing hormone-releasing hormone (LHRH) agonist] were continued during the COVID-19 pandemic since they do not affect the immune system (55). In terms of radiotherapy, Leonardi et al. reported that there was no significant difference in the time interval between treatments and radiotherapy for high-risk patients (56).

### Cluster 5: Clinical vaccine management of breast cancer patients during the pandemic

Vaccination is an essential step in the fight against this devastating pandemic and is relatively safe for breast cancer patients. Can people using CDK 4/6 inhibitors be vaccinated, and what is the effect? The answer is that vaccination is available. Patients with breast cancer who underwent the treatment of CDK4/6 inhibitors developed SARS-CoV-2 NAbs in response to the first dose of COVID-19 vaccines, similar to the general population (57, 58). It is also worth noting that overdiagnosis should be avoided in breast cancer patients who develop lymphadenopathy (LAP) after vaccination. LAP related to COVID-19 vaccine tended to show increased cortical thickness without cortical irregularity, showing some suspicious features more often than others and persisting longer than anticipated (59). Accordingly, the recommendation for breast cancer patients about to undergo surgery is that the vaccination is given before or 1 week after surgery (60). The above findings from clinical studies suggest that vaccine-related adverse events are low and most of them have a short duration in cancer patients, that no serious adverse events directly related to the vaccine have been observed, and that the benefits of the vaccine may far outweigh the vaccine-related harms (61).

### Limitations

Although bibliometric analysis and visualization methods were initially employed for the evaluation of the quality and quantity of research BCMP in this study, it also had some limitations. First, the bibliometric analysis only included a single database for search. Second, we only searched the titles, and there may be distribution articles missing. Third, burst keywords analysis cannot be performed due to the publication of the literature from 1 January 2020 to 1 April 2022. Despite the above limitations, our analysis can provide a reference for the research characteristics of BCMP.

## Conclusion

Bibliometric techniques were employed for examining publications, research hotspots, and trends in breast cancer management during the pandemic. The findings of this study reveal that the United States of America, Italy, and China have made substantial contributions to the number of publications, institutions, magazines, and citations, which has facilitated the development of BCMP. The Buonomo-centered team, University of Rome Tor Vergata, and the *Breast Cancer Research and Treatment* journal were the most prolific in the field. Furthermore, hotspots and trends in research are screening, surgery, rehabilitation, emotion, diagnostic treatment, and vaccine management of breast cancer during the pandemic. As more insights are gained into COVID-19, breast cancer management is ever-changing and requires ongoing research and conclusion.

## Data availability statement

The original contributions presented in the study are included in the article/**Supplementary Material**. Further inquiries can be directed to the corresponding authors.

## Author contributions

Writing original draft: P-fL, J-tL, and TD. Validation: J-tL and TD. Investigation: P-mF and G-XL. Methodology: J-tL and TD. Software: P-fL. Supervision: X-CC. Project administration: P-fL. Data interpretation: Ping-ming Fan. Review and editing: G-XL, X-CC, and P-mF. All authors contributed to the article and approved the submitted version.

## Acknowledgments

The author G-XL wishes to acknowledge the financial support of the “Xiamen Health High-Level Talent Training Program”.

## Conflict of interest

The authors declare that the research was conducted in the absence of any commercial or financial relationships that could be construed as a potential conflict of interest.

## Publisher’s note

All claims expressed in this article are solely those of the authors and do not necessarily represent those of their affiliated organizations, or those of the publisher, the editors and the reviewers. Any product that may be evaluated in this article, or claim that may be made by its manufacturer, is not guaranteed or endorsed by the publisher.
