# Supplementary material for: Research trends and hotspots of breast cancer management during the COVID-19 pandemic: A bibliometric analysis
Source: Front Oncol. 2022 Aug 3;12:918349. doi: 10.3389/fonc.2022.918349 (PMC9381881; doi:10.3389/fonc.2022.918349)
Supplement: Supplementary file 1 [file Table_1.doc]

**Table S1 Top 10 cited articles in the field of BCMP**

| **Rank** | **Article Title** | **Author** | **Journal** | **IF** | **Citation** | **Country** |
| --- | --- | --- | --- | --- | --- | --- |
| 1 | Recommendations for triage, prioritization and treatment of breast cancer patients during the COVID-19 pandemic | Curigliano et al | BREAST | 4.38 | 110 | Italy |
| 2 | Breast Cancer and COVID-19: The Effect of Fear on Patients' Decision-making Process | Vanni, G et al | IN VIVO | 2.15 | 63 | Italy |
| 3 | Patient-reported treatment delays in breast cancer care during the COVID-19 pandemic | Papautsky et al | BREAST CANCER RESEARCH AND TREATMENT | 4.87 | 54 | USA |
| 4 | COVID-19 in breast cancer patients: a cohort at the Institute Curie hospitals in the Paris area | Vuagnat et al | BREAST CANCER RESEARCH | 6.48 | 51 | France |
| 5 | Patient-reported Outcomes of Patients with Breast Cancer During the COVID-19 Outbreak in the Epicenter of China: A Cross-sectional Survey Study | Li, JJ et al | CLINICAL BREAST CANCER | 3.22 | 47 | China |
| 6 | COVID-19 Lockdown and Its Adverse Impact on Psychological Health in Breast Cancer | Swainston et al | FRONTIERS IN PSYCHOLOGY | 2.98 | 35 | England |
| 7 | The COVID-19 pandemic and impact on breast cancer diagnoses: what happened in England in the first half of 2020 | Gathani, T et al | BRITISH JOURNAL OF CANCER | 7.64 | 28 | England |
| 8 | Healthcare utilization among breast cancer patients during the COVID-19 outbreak | Shinan-Altman S et al | PALLIATIVE & SUPPORTIVE CARE | 2.26 | 27 | England |
| 9 | Assessing the Impact of the COVID-19 Outbreak on the Attitudes and Practice of Italian Oncologists Toward Breast Cancer Care and Related Research Activities | Poggio, F et al | JCO ONCOLOGY PRACTICE | 3.55 | 26 | Italy |
| 10 | The Effect of Coronavirus (COVID-19) on Breast Cancer Teamwork: A Multicentric Survey | Vanni, G et al | IN VIVO | 2.15 | 24 | Italy |
