# Supplementary material for: Research trends and hotspots of breast cancer management during the COVID-19 pandemic: A bibliometric analysis
Source: Front Oncol. 2022 Aug 3;12:918349. doi: 10.3389/fonc.2022.918349 (PMC9381881; doi:10.3389/fonc.2022.918349)
Supplement: Supplementary file 2 [file Table_2.doc]

**Table S2 Clusters of the top five high-frequency keywords.**

| Cluster 0 | Cluster 1 | Cluster 2 | Cluster 3 | Cluster 4 | Cluster 5 |
| --- | --- | --- | --- | --- | --- |
| mammography | surgery | ACE2 | quality of life | telemedicine | CDK46 |
| Screening | Knowledge | recovery | anxiety | endocrine | Vaccination |
| delay | Pandemic | resilience | insomnia | neoadjuvant | hesitancy |
| Screening suspension | Incidence | telerehabilitation | stress | chemotherapies | management |
| Anxiety | Breast cancer | exercise | fear recurrence | tele-oncology | staging |
